# Utilization, reimbursement, and price trends for Hepatitis C virus medications in the US Medicaid programs: 2001–2021

**DOI:** 10.1016/j.rcsop.2023.100383

**Published:** 2023-11-28

**Authors:** Musaab H. Gari, Abdulrahman Alsuhibani, Amin Alashgar, Jeff J. Guo

**Affiliations:** a James L. Winkle College of Pharmacy, University of Cincinnati Academic Health Center, Cincinnati, OH 45267, USA; b Department of Pharmacy Practice, Unaizah College of Pharmacy, Qassim University, Saudi Arabia

**Keywords:** Hepatitis C virus, Direct acting antivirals, Specialty medications, Medicaid, Utilization, Reimbursement, Price, Policy

## Abstract

**Background:**

Hepatitis C Virus (HCV) remains a challenging health problem worldwide, with increasing incidence despite being curable with Direct Acting Antiviral (DAA) agents.

**Objective:**

This study aimed to describe the utilization, reimbursement, and price trends of HCV treatments and evaluate the influence of treatment guidelines and policies.

**Methods:**

A retrospective, descriptive drug utilization study conducted using the outpatient pharmacy data extracted from the Centers for Medicaid and Medicare Services State Drug Utilization Data between 2001 and 2021. All HCV treatments approved in the US were included, conventional therapy (CT), and DAA agents. The annual secular trends were calculated for each medication's total number of prescriptions, reimbursements, and prices. The average reimbursement per prescription was calculated and utilized as a proxy of prices. The HCV treatment guideline and policies and legislation were evaluated overtime to measure the impact on the trends.

**Results:**

Despite CT having a higher total utilization, DAA agents commanded significantly greater reimbursements, with 4.1 billion USD for CT and 19.45 billion USD for DAA agents. CT utilization increased rapidly and dominated the market until 2011, peaking at 379,696 prescriptions in 2003 but declining afterward. DAA agents' utilization increased rapidly in their first year: i.e., sofosbuvir reached 50,377 prescriptions with 1.3 billion USD in 2014, while ledipasvir/sofosbuvir reached 79,387 prescriptions with 2 billion USD in 2015. The average price per prescription was high for the DAA agents, like 24,992 USD for sofosbuvir and 22,787 USD for ledipasvir/sofosbuvir, compared to CT medications ribavirin, around 500 USD, and pegINF, around 3000 USD. The new DAA agents replaced CT, and initiating market competition among DAA agents.

**Conclusion:**

The introduction of multiple DAA agents slightly changed their prescription prices but remained high during the study period. The recent increase in HCV incidence cases indicates accessibility issues for costly and effective DAA agents, with treatment guidelines and policies playing a critical role in shaping these trends.

## 1 Introduction

The Hepatitis C Virus (HCV) has undergone a significant shift in treatment options in recent years, with the discovery of Direct Acting Antiviral (DAA) agents leading to the disease becoming a curable condition. Nonetheless, HCV remains a prevalent cause of liver diseases globally. Estimating 58 million people live with chronic HCV infection with the potential of a range of adverse health outcomes if left untreated.^1^ In the United States, the Centers for Disease Control and Prevention (CDC) reported a 124% increase in the incidence rate of acute HCV infections since the advent of DAA agents in 2013, with an estimated 66,700 infected cases in 2020.^2^ This highlights the ongoing importance of HCV as a public health concern and the need for continued efforts to improve diagnostic and treatment options for those affected.

Conventional therapy (CT), became the standard of care in 2001, which includes ribavirin plus pegylated interferon (pegINF) α-2a, but had limited efficacy and tolerability compared to DAA agents.^3^ In 2014, the HCV Guidance Panel developed a clinical guideline to manage the treatment options, which resulted from a collaborative effort between the American Association for the Study of Liver Diseases (AASLD) and the Infectious Diseases Society of America (IDSA). The guideline is regularly updated and accessible online, providing easy access to testing, treatment, and management recommendations for various populations based on factors such as the patient's HCV status and virus genotype.^4^ Since the inception of the guideline, multiple versions have been published to reflect the latest updates in HCV therapy. Thus, utilizing DAA agents is subject to changes in the guideline, as newer agents with superior results are approved. Several DAA agents showed initial promising efficacy but have since been discontinued due to decreased utilization as newer, more effective agents became available (Table 1). However, challenges such as high treatment prices, gaps in diagnosis and treatment, and limited proactive prevention programs can limit accessibility for specific populations, underscoring the need for continuous evaluation and adaptation of treatment strategies to optimize patient care and resource allocation.

**Table 1:** Brand & generic names, manufacturers, FDA approval date, expected patent expirations for HCV medications of pegINF, ribavirin, and DAA agents used by US Medicaid program from 2001 to 2021.

Brand name	Generic name	Manufacture company	FDA approval	Patent Exp.	D/C
**Conventional Therapy**					
Rebetol Ribasphere Copegus Moderiba	Ribavirin	Genentech Merck Sharp & Dome Kadmon Pharms	06/03/1998	2023	Copegus Moderiba

Pegasys	Peginterferon alfa-2a	Hoffmann-La Roche Inc.	12/20/2002	2018	

PEG-Intron	Peginterferon alfa-2b	Merck & Co., Inc.	12/28/2001	2013	09/2021


**Direct Acting Antivirals**					
Victrelis	Boceprevir	Schering Corporation - Merck	05/13/2011	2027	12/31/2015

Incivek	Telaprevir	Vertex Pharmaceuticals Incorporated	05/23/2011	2025	10/16/2014

Olysio	Simeprevir	Janssen Pharmaceuticals Inc.	11/22/2013	2029	05/25/2018

Sovaldi	Sofosbuvir	Gilead Sciences Inc	12/06/2013	2028	

Harvoni	Ledipasvir - Sofosbuvir	Gilead Sciences Inc	10/10/2014	2030	

Viekira	Ombitasvir - Paritaprevir - Ritonavir - Dasabuvir	Abbvie	12/19/2014	2032	1/1/2019

Technivie	Ombitasvir - Paritaprevir - Ritonavir	Abbvie	07/24/2015	2032	1/1/2019

Daklinza	Daclatasvir	Bristol-Myers Squibb Company	07/24/2015	2027	1/4/2019

Zepatier	Elbasvir - Grazoprevir	Merck Sharp & Dohme	01/28/2016	2031	

Epclusa	Sofosbuvir - Velpatasvir	Gilead Sciences Inc	06/28/2016	2028	

Vosevi	Sofosbuvir - Velpatasvir - Voxilaprevir	Gilead Sciences Inc	07/18/2017	2028	

Mavyret	Glecaprevir - Pibrentasvir	Abbvie and Enanta Pharmaceuticals	08/03/2017	2030	

The specialty medications, including the DAA agents, are characterized by unique features, such as high prices, complex disease management, and manufacturer restrictions.^5^ Despite manufacturer discounts and rebates, these drugs' prices have continuously increased over the years.^6^ Access to specialty medications is crucial since they are often prescribed for severe conditions.^7^ Recently, multiple government efforts were made to reduce prescription prices. The Centers for Medicare & Medicaid Services (CMS) proposed the International Pricing Index (IPI) in 2018 as part of the Blueprint to Lower Drug Prices, which former President Trump supported.8., 9., 10. The IPI was criticized, and the drug pricing rules were temporarily blocked in January 2021.11., 12., 13. The Inflation Reduction Act of 2022 replaced the IPI. It empowered government-based plans to limit drug price increases, negotiate pricing, and expand coverage. From 2022 to 2031, drug manufacturers must pay rebates to CMS if medication prices rise above the inflation rate, and CMS will also negotiate the prices of 10 high-cost medications from 2026 and reach 20 medications by 2029.14., 15, 16

The literature on HCV treatment trends among Medicaid beneficiaries is limited, with only a single study from 2018 analyzing three years of DAA agent utilization, where all agents were discontinued except for sofosbuvir and ledipasvir/sofosbuvir.^17^ Understanding these trends is crucial, as inadequate access to treatment can escalate public health burdens, including increased HCV transmission and higher long-term healthcare prices. Furthermore, while more expensive DAA agents often yield better patient outcomes, their high prices can strain Medicaid budgets and limit treatment accessibility.^18^, ^19^, ^20^, ^21^ Certain DAA treatments within the Medicaid beneficiary population may be more accessible or preferred due to treatment guidelines, policies, price, or eligibility criteria considerations.^22^ Thus, this study aims to bridge the knowledge gap by comprehensively examining HCV treatments' utilization, reimbursement, and price trends over the past two decades while evaluating the influence of treatment guidelines and policies on them, offering valuable insights into HCV treatment for Medicaid beneficiaries.

## 2 Methods

### 2.1 Study design

A retrospective, descriptive data analysis study was conducted to evaluate the utilization of HCV treatments approved by the US Food and Drug Administration (FDA) from the first quarter of 2001 through the fourth quarter of 2021. This analysis incorporated a comprehensive review of national pharmacy data files sourced from CMS to examine prescription patterns and total pharmacy reimbursements under Medicaid for each HCV medication.

### 2.2 Data source

Medicaid reimbursement for medications encapsulates the fiscal transactions whereby Medicaid pays pharmacies for dispensing prescription drugs to its beneficiaries. The CMS meticulously documents these transactions quarterly for beneficiaries across all states and the District of Columbia, ensuring a robust dataset with broad coverage that reflects the national utilization trends. The longitudinal nature of the dataset allows for an analysis of trends over a significant period, aligning with the duration of the introduction and evolution of DAA treatments. The extracted data were distinguished by the National Drug Code (NDC), a unique 11-digit identifier assigned to each drug, facilitating a granular analysis of medication-specific trends. To facilitate this data, the pre-rebate totals from the National Summary Files were combined across all states' databases.^23^

### 2.3 Data collection and assessments

The study incorporated data from both Conventional Therapies (pegINF-α2a, pegINF-α2b, and ribavirin) and all twelve DAA agents approved for HCV treatment.^24^ Aggregation of the data involved summing the total number of prescriptions and their corresponding reimbursement amounts in US dollars (USD). For comparative purposes, the pricing per prescription was computed by dividing the total reimbursement by the total number of prescriptions. The consolidation of data of pegINF-α2a, pegINF-α2b, and ribavirin enabled a comparative analysis between CT and DAA agents. The total reimbursements for CT and DAA were normalized for inflation using the Consumer Price Index (CPI) adjusted to the fiscal context of 2021, which allowed an accurate longitudinal financial analysis.^25^ Furthermore, an examination of the impact of treatment guidelines and policy shifts was conducted by correlating these events with alterations in prescription distributions.

Descriptive statistical methods were employed to assess the trends in number of prescriptions, overall reimbursement amount, and the price per prescription throughout the last two decades. The market share of HCV treatments within the Medicaid program was quantified, both as a percentage of prescription counts and reimbursement totals. Abnormalities identified within the CMS database for the years 2006 and 2007 were excluded from the analysis, given their negligible effect on the primary aims and findings of the study, particularly considering that DAA agents were introduced post-2011. Analytical processes were conducted using SAS Version 9.4 (SAS Institute Inc., Cary, NC, USA) and Microsoft Excel 2023 Version 16.70 (Microsoft Corporation, Redmond, WA, USA).

## 3 Results

### 3.1 Evolution of CT and DAA prescriptions

**Fig. 1:**
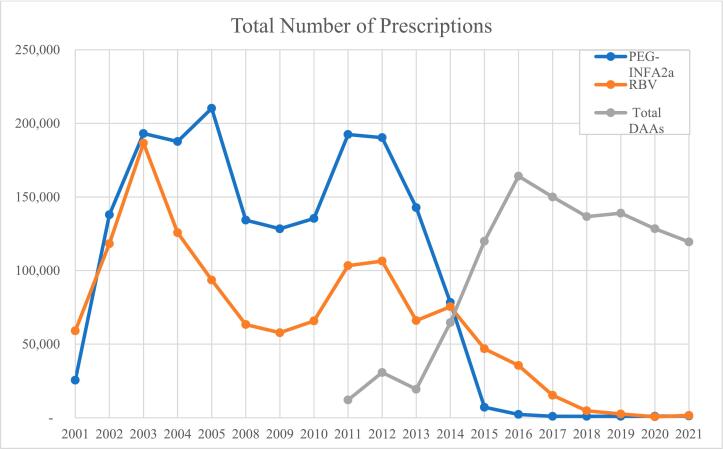
Annual secular trends of total number of prescriptions for pegINF, ribavirin, and DAA agents in the US Medicaid Programs 2001–2021.

The analysis of prescription trends within the Medicaid program from 2001 to 2011 reveals a dynamic shift in the therapeutic landscape for HCV treatment. Initially, CT, consisting predominantly of interferon-based treatments, dominated the market with its utilization peaking at 379,696 prescriptions in 2003. From 2004 onwards, while the utilization of RBV showcased a steady decline, pegINF briefly increased in 2005 before beginning a gradual decline. The lowest utilization for CT was observed in 2009, with 186,200 prescriptions. The approval of the first DAA agents, boceprevir and telaprevir, initiated a downward trend in CT utilization. The subsequent approval of more DAA agents led to a further decrease in CT use, indicating an inverse relationship between the utilization of CT and DAA agents (Fig. 1).

### 3.2 Market dynamics

Since 2011, twelve DAA agents have entered the market, though only six currently remain available (Table 1). The entry of new DAA agents resulted in significant market competition, observed as fluctuations in market share represented by the number of prescriptions. In 2014, the first single pill combinations were approved are ledipasvir/sofosbuvir and ombitasvir/paritaprevir/ritonavir/dasabuvir. A remarkable market penetration was observed with ledipasvir/sofosbuvir reflected by 79,387 prescriptions in 2015, contrasted with ombitasvir/paritaprevir/ritonavir/dasabuvir, which secured only 9004 prescriptions, diminishing to 561 by 2018 (Table 2).

**Table 2:** Utilization market-share percentages of daa agents and their average prescription prices in the US Medicaid Programs (2011−2021).

Year	Victrelis	Incivek	Olysio	Sovaldi	Harvoni	Viekira	Technivie	Daklinza	Zepatier	Epclusa	Vosevi	Mavyret
2011	3231 (11.75%)	8843 (23.69%)										

2012	13,146 (47.79%)	17,613 (47.19%)										

2013	9396 (34.16%)	9850 (26.39%)	2 (0.02%)	136 (0.13%)								

2014	1646 (5.98%)	861 (2.31%)	8736 (80.38%)	50,377 (48.20%)	3039 (1.28%)							

2015	70 (0.25%)	125 (0.33%)	1722 (15.84%)	27,109 (25.94%)	79,387 (33.51%)	9004 (32.04%)	136 (41.85%)	2362 (13.96%)				

2016	19 (0.07%)	33 (0.09%)	323 (2.97%)	25,182 (24.09%)	81,250 (34.30%)	13,269 (47.21%)	152 (46.77%)	13,528 (79.94%)	14,901 (22.01%)	15,513 (6.01%)		

2017			86 (0.79%)	1533 (1.47%)	44,121 (18.62%)	5271 (18.75%)	37 (11.38%)	978 (5.78%)	43,347 (64.03%)	43,844 (16.99%)	1064 (9.17%)	9734 (3.42%)

2018				110 (0.11%)	11,216 (4.73%)	561 (2.00%)		55 (0.33%)	4462 (6.59%)	24,446 (9.48%)	3482 (30.01%)	92,331 (32.42%)

2019				23 (0.02%)	5072 (2.14%)				1699 (2.51%)	50,003 (19.38%)	2549 (21.97%)	79,661 (27.97%)

2020				26 (0.02%)	9675 (4.08%)				2119 (3.13%)	62,670 (24.29%)	2332 (20.10%)	51,605 (18.12%)

2021				27 (0.03%)	3138 (1.32%)				1175 (1.74%)	61,529 (23.85%)	2177 (18.76%)	51,450 (18.07%)

Avg. Rx Price	4780 USD	16,135 USD	20,579 USD	24,992 USD	22,787 USD	21,356 USD	23,965 USD	16,132 USD	10,268 USD	16,881 USD	22,350 USD	12,094 USDf

### 3.3 Economic implications

**Fig. 2:**
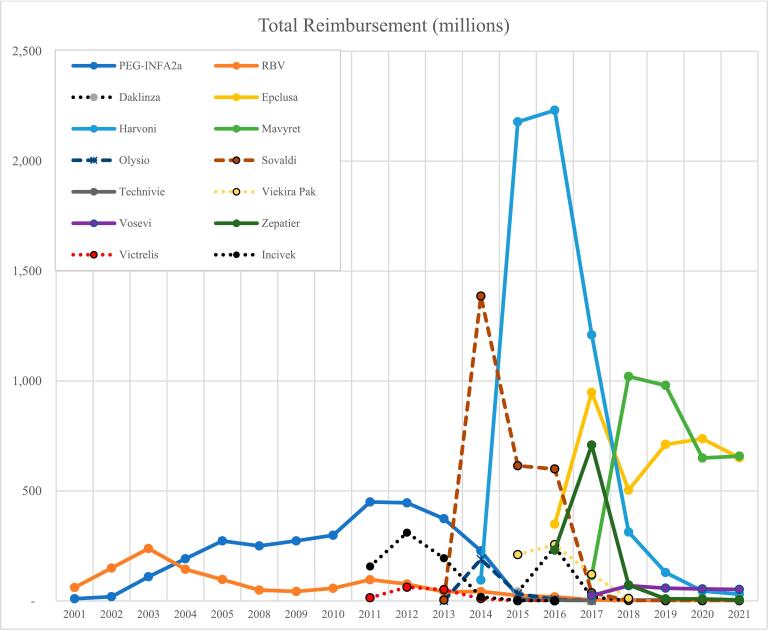
Annual secular trends of total reimbursement for pegINF, ribavirin, and DAA agents in the US Medicaid Programs 2001–2021.

The fiscal analysis of HCV treatment reimbursement patterns uncovers a stark contrast between CT and DAA agents. Despite the higher prescription volume of CT, DAAs commanded disproportionately higher reimbursements, amassing 19.45 billion USD compared to CT's 4.1 billion USD. When adjusting for inflation to the year 2021, the financial disparity persisted, with DAA agents incurring total reimbursements of 21.5 billion USD versus 5.2 billion USD for CT.^25^ The annual reimbursements for pegINF agents averaged around 156 million USD from 2001 to 2021, whereas ribavirin agents secured merely 24.5 million USD. Post-2013, reimbursements for CT agents declined, while DAA agents marked higher reimbursements for the individual products. For instance, simeprevir and sofosbuvir received reimbursements of 189 million USD and 1.3 billion USD in 2014, respectively. Similarly, ledipasvir/sofosbuvir recorded reimbursements of over 2 billion USD in both 2015 and 2016, and glecaprevir/pibrentasvir received a reimbursement of 1.02 billion USD in 2018 (Fig. 2).

### 3.4 Prescription pricing

**Fig. 3:**
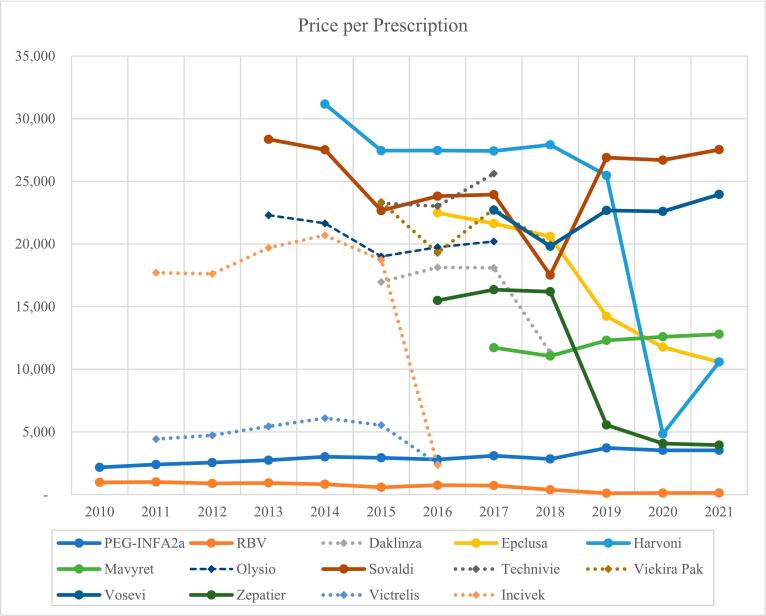
Annual secular trends of price per prescription for pegINF, ribavirin, and DAA agents in the US Medicaid Programs 2010–2021.

A critical examination of the prescription pricing trends for HCV medications indicates an initial price surge for DAAs following their approval. Subsequent years, however, witnessed substantial price reductions for agents such as ledipasvir/sofosbuvir, elbasvir/grazoprevir, and sofosbuvir/velpatasvir between 2018 and 2021. In contrast, the prices for sofosbuvir and glecaprevir/pibrentasvir have been on an uptrend in the recent years. Meanwhile, the cost trajectory for pegINF and ribavirin has been mixed, with a modest increase for pegINF and a decline for ribavirin (Fig. 3).

## 4 Discussion

The study revealed that after CT became the standard of care for HCV in 2001, its utilization peaked in 2003, but subsequently declined due to adverse effects and treatment inefficacies.^26^^,^^27^ The FDA approval of the first DAA agents, boceprevir and telaprevir, as a combination therapy to CT, increased CT's utilization by 47% between 2010 and 2012.^24^ Nevertheless, as newer and more efficacious DAA agents like Simeprevir and sofosbuvir became available, they supplanted the older DAAs, leading to their eventual phase-out from the market.^28^, ^29^, 30., 31. The approval of ledipasvir/sofosbuvir in 2015, the first single-pill HCV treatment, further shifted the treatment paradigm, evidenced by its rapid market capture and the concurrent decline in the use of CT and older DAA agents.^32^ The healthcare market witnessed a brand-brand competition where the therapeutic profiles of DAAs agents through their efficacy and patient tolerance became the primary determinants for market success. Therefore, all the six DAA agents that were discontinued from the market were to decreased utilization.

The study found a dichotomy in pricing dynamics while examining the trends in prescription prices for HCV treatment. The prices for pegINF products saw a 339% increase from 2001 to 2021, whereas ribavirin prices dropped by 87%. The DAA agents, despite price fluctuations, consistently overshadowed CT in price, with discontinued DAAs generally reducing their prices pre-discontinuation. The emergence of newer DAAs catalyzed market and price competitions, exemplified by elbasvir/grazoprevir's 65.7% price drop within a single year.

The use of DAA agents for treating HCV is considered cost-effective, cost-saving, and lead to better clinical outcomes compared to delayed treatment.^18^, ^19^, ^20^, ^21^ These agents not only offer immediate therapeutic advantages but also carry the potential to mitigate long-term health consequences such as advanced liver disease and the need for liver transplantation. Moreover, the recent efforts to relaxing the eligibility criteria, such as advanced liver fibrosis, abstinence from substance use, and consultation with a specialist medical provider, for DAAs treatment for Medicaid program is beneficial to enhance treatment accessibility.^22^

The study underscores the significance of HCV treatment guidelines in directing treatment trends towards more effective and patient-centric options. For instance, the 2015 update recommended sofosbuvir in combination with CT and available DAA agents tailored to genotype and patient characteristics, fostering a shift in therapeutic approch.^33^ Subsequent changes in 2018 deprioritized the use of medications like pegINF, simeprevir, ombitasvir/paritaprevir/ritonavir, ombitasvir/paritaprevir/ritonavir/dasabuvir, or daclatasvir while emphasizing newer DAA agents like glecaprevir/pibrentasvir for most patients.^34^ The 2019 update further refined these recommendations by providing detailed information on patient characteristics to be considered in treatment decisions.^35^ These guidelines updated steered clinicians away from certain treatments and towards adopting newer therapies with potentially better efficacy and tolerability. Consequently, these updates likely influenced utilization trends, as evidenced by the discontinuation of specific DAA agents and the increased utilization of alternatives like glecaprevir/pibrentasvir.

The Inflation Reduction Act of 2022 aims to provide a legislative framework for addressing the high prices of DAA agents. However, pharmaceutical manufacturers and public health entities have launched initiatives to mitigate high prices and enhance access. In developing countries, Gilead Sciences, Inc. and Bristol-Myers Squibb have permitted the production of generic DAA agents at lower prices to promote treatment accessibility.^36^, 37., 38 Meanwhile, in the US, the Louisiana Department of Health and Department of Corrections partnered with Asegua Therapeutics LLC in 2019 to provide unrestricted access to generic sofosbuvir/velpatasvir through a subscription model with capped annual costs, providing treatment access to 11,000 patients at no cost in 2022 and aiming to treat 31,000 patients by 2024.^39^^,^^40^ These efforts resulted in a 151.7% increase in sofosbuvir/velpatasvir utilization among Medicaid beneficiaries, and the average prescription price decreased by 48.6% from 20,595 USD in 2018 to 10,576 USD in 2021, demonstrating the program's effectiveness in addressing high medication prices and improving treatment accessibility while maintaining high reimbursements between 2019 and 2021. Policies and initiatives aimed at reducing costs and increasing access have already shown promising results. The continued implementation and potential expansion of such strategies are likely to sustainably impact patient access and the broader pharmaceutical landscape, potentially leading to more innovative and cost-effective HCV treatment solutions.

Factors that influence drug prices include research and development costs, manufacturing costs, and granted market exclusivity.^7^^,^^41^ Although research and development costs for DAA agents can be substantial, government funding typically sponsors them, such as Gilead Sciences, Inc.'s acquisition of sofosbuvir during Phase III clinical trial, which was already funded with over 1 billion USD from the National Institutes of Health (NIH).^42^^,^^43^ Although manufacturing costs were used to justify high drug prices, Sofosbuvir's estimated manufacturing costs for a 12-week course in 2014 were only 67–136 USD.^44^ However, high drug prices are primarily due to government-granted exclusivity and free-market pricing, with medication prices in the US 256% higher in 2018 than 32 other countries combined, and the average net price of brand-name medications in Medicaid increasing from 147 to 218 USD between 2009 and 2018.^45^^,^^46^ Granted market exclusivity and patent protection play a pivotal role in drug pricing in the US. However, the policies and initiatives would help mitigate the excessive high prices and motivate pharmaceutical manufacturers to improve medication accessibility and affordability.

This research has several strengths, including its extended duration, comprehensive list of medications, and substantial number of prescriptions derived from multiple states in the US. Nevertheless, certain limitations must be considered when interpreting the results. The data from the CMS database lack patient-specific details, precluding assessments of treatment success or failure and potentially introducing a selection bias based on unobserved patient demographics or provider preferences. Time-related biases may also be present, as changes in healthcare policies and drug approvals over two decades could influence observed trends. The aggregation of quarterly data into yearly figures and the combination of multiple brand names for CT might mask granular variations. Additionally, the findings are specific to Medicaid's low and middle-income beneficiaries, limiting external validity and generalizability to other populations or healthcare systems. Thus, interpreting the results necessitates caution and an understanding of these limitations.

## 5 Conclusion and relevance

The advent of DAA agents has precipitated a marked increase in Medicaid expenditure, escalating from an annual $100 million to a range of $1–2 billion. While the clinical benefits of these treatments are substantial and may justify the expense, it is imperative to dissect cost and utilization trends to ensuring equitable access and optimizing healthcare budgets. The treatment guideline, regularly updated to reflect the emerging evidence, thereby modulating DAA use. Although policies did not directly impact the utilization trends, they harbor the potential to mitigate the steep prices of DAAs and incentivize the pharmaceutical sector towards enhancing access to treatment. Further research is essential to evaluate new policies' impact and to develop more effective strategies, such as preventive measures, in the management of HCV.

## Declaration of Competing Interest

The authors declare the following financial interests/personal relationships which may be considered as potential competing interests:

No funding was received for conducting this study. The authors have no relevant financial or non-financial interests to disclose.
